# Does the climate warming hiatus exist over the Tibetan Plateau?

**DOI:** 10.1038/srep13711

**Published:** 2015-09-02

**Authors:** Anmin Duan, Zhixiang Xiao

**Affiliations:** 1 State Key Laboratory of Numerical Modelling for Atmospheric Sciences and Geophysical Fluid Dynamics (LASG), Institute of Atmospheric Physics (IAP), Chinese Academy of Sciences (CAS), Beijing 100029, China; 2 Collaborative Innovation Center on Forecast and Evaluation of Meteorological Disasters, Nanjing University of Information Science & Technology, Nanjing 210044, China; 3 University of Chinese Academy of Sciences, Beijing 100049, China

## Abstract

The surface air temperature change over the Tibetan Plateau is determined based on historical observations from 1980 to 2013. In contrast to the cooling trend in the rest of China, and the global warming hiatus post-1990s, an accelerated warming trend has appeared over the Tibetan Plateau during 1998–2013 (0.25 °C decade^−1^), compared with that during 1980–1997 (0.21 °C decade^−1^). Further results indicate that, to some degree, such an accelerated warming trend might be attributable to cloud–radiation feedback. The increased nocturnal cloud over the northern Tibetan Plateau would warm the nighttime temperature via enhanced atmospheric back-radiation, while the decreased daytime cloud over the southern Tibetan Plateau would induce the daytime sunshine duration to increase, resulting in surface air temperature warming. Meanwhile, the *in situ* surface wind speed has recovered gradually since 1998, and thus the energy concentration cannot explain the accelerated warming trend over the Tibetan Plateau after the 1990s. It is suggested that cloud–radiation feedback may play an important role in modulating the recent accelerated warming trend over the Tibetan Plateau.

The global average temperature warmed by 0.85 °C in the period 1880–2012, with 1983–2012 being the warmest 30-year period of the last 1400 years in the Northern Hemisphere^1^. However, Easterling *et al*.^2^ demonstrated that for the period 1998–2008, there was no real warming of the global temperature. Since then, many studies^3^,^4^,^5^ have discussed the slowdown in global warming. The potential causes have been ascribed to natural variability^4^,^6^, reduced radiative forcing^7^,^8^, coverage bias in observations^9^, and a smaller warming response to atmospheric carbon dioxide concentrations^10^,^11^.

The Tibetan Plateau is the highest plateau in the world, and exerts a significant influence on regional and global atmospheric circulation^12^,^13^,^14^,^15^,^16^, in particular serving as “the world water tower”^17^. The Tibetan Plateau is regarded as a key region for global climate change^18^,^19^. Therefore, it is pivotal to understand the climate change of the plateau. The land surface temperature warming process, diurnal variations and surface heat fluxes over the Tibetan Plateau have been revealed through remote sensing techniques in many previous studies^20^,^21^,^22^; and from this work, we know that the Tibetan Plateau surface air temperature has increased at a faster rate than the warming rate in the Northern Hemisphere^22^. Previous studies^23^,^24^ have also found that the surface air temperature of the plateau increased at a faster rate during 1951–2004 and 1961–2006 than in the surrounding regions of lower elevation, especially during winter. The climate warming over the Tibetan Plateau further results in a consequence of increasing climate aridity^25^. But how does the situation over the Tibetan Plateau compare to the global warming hiatus during the last decade, and is it different to other parts of China? If so, what are the possible reasons? In this work, we address these issues by comparing the temperature change between the two periods 1980–1997 and 1998–2013 over the Tibetan Plateau and the surrounding areas based on historical observations.

## Results

**Figure 1:**
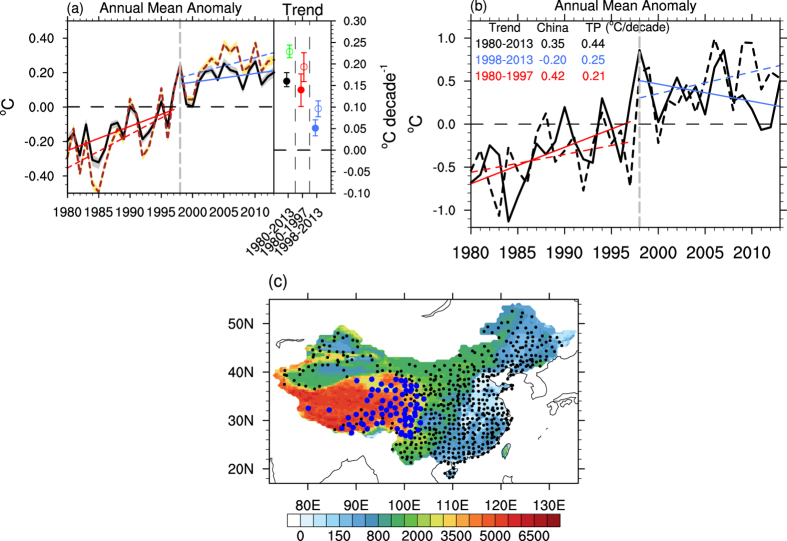
Global and China annual mean surface air temperature anomaly series during 1980–2013, relative to the base period of 1981–2010. In (**a**) solid curves indicate global temperature, and dashed curves indicate the Northern Hemisphere temperature. The grey and yellow shaded areas indicate the uncertainty ranges obtained from the 100 members of HadCRUT4. The solid circles represent the global temperature trend, and the open circles show the Northern Hemisphere trend, the error bars show the range of the trend obtained from the 100 members. Panel (**b**) as (**a**), except for China (solid lines) and the Tibetan Plateau (dashed lines). Panel (**c**) shows the distribution of the meteorological stations used in this paper. The shaded area indicates the terrain height (units: m) and the figure is created by open access software of NCAR Command Language (NCL).

Figure 1a shows the global and Northern Hemisphere annual mean temperature trend during 1980–2013. During this period, the Northern Hemisphere is characterized by a larger warming trend (0.23 °C decade^−1^) than the global mean (0.16 °C decade^−1^). Since 1998, however, both the global and Northern Hemisphere mean temperatures show only a small warming trend: 0.05 °C decade^−1^ during 1998–2013 globally and 0.10 °C decade^−1^ for the Northern Hemisphere. Such a result is consistent with that reported by the IPCC^1^.

Under the background of global warming, the surface air temperature over both the Tibetan Plateau and China increased quickly during 1980–2013, with an even larger amplitude compared to the mean case for the Northern Hemisphere: 0.44 °C decade^−1^ for the 73-station average over the Tibetan Plateau and 0.35 °C decade^−1^ for the 563-station average in other parts of China (Fig. 1b). These results are similar to previous findings that the warming is more rapid in China and the Tibetan Plateau than is found globally^23^. At the first stage (1980–1997), the warming amplitude is 0.42 °C decade^−1^ in other parts of China, about twice the warming rate over the Tibetan Plateau (0.21 °C decade^−1^). At the second stage (1998–2013), however, the warming rates in other parts of China have an opposite cooling trend (−0.20 °C decade^−1^), while the surface air temperature over the Tibetan Plateau exhibits an accelerated warming trend (0.25 °C decade^−1^), undocumented in previous studies.

**Figure 2:**
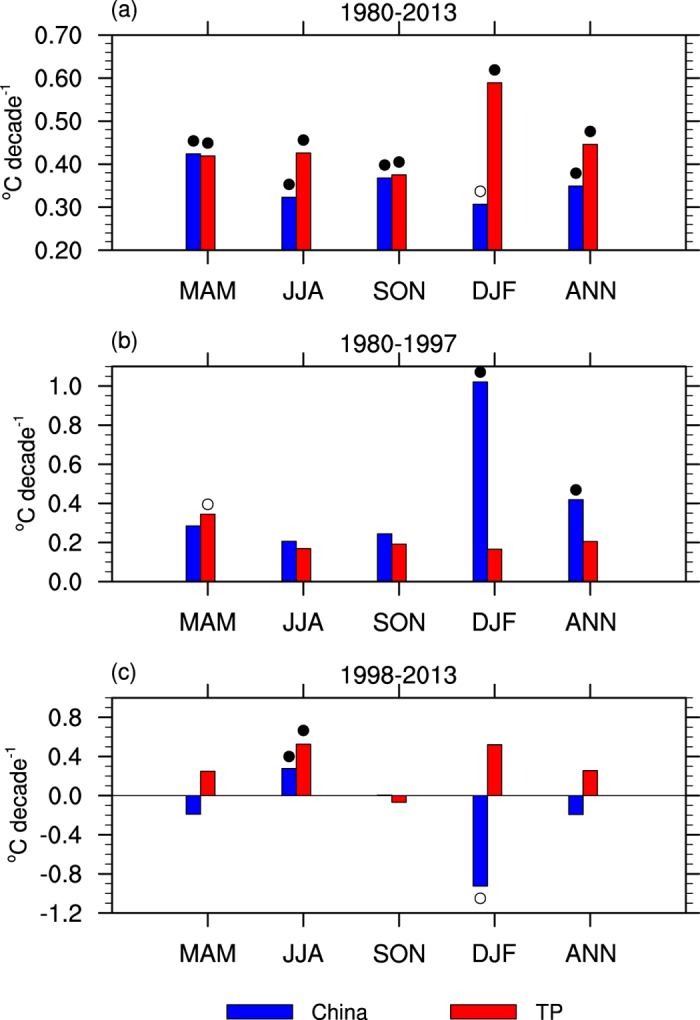
The annual and seasonal mean temperature trend over China (blue) and the Tibetan Plateau (TP, red) based on station data in spring (March–April–May, MAM), summer (June–July–August, JJA), autumn (September–October–November, SON), winter (December–January–February, DJF), and annual (ANN). Units: °C decade^−1^. The open (filled) circles indicate the trend is significant at 90% (95%) confidence level, according to a two-tailed Student’s *t*-test. The figures were produced using NCL.

In order to compare the seasonal and regional differences of temperature change over the Tibetan Plateau and other parts of China, Fig. 2 shows the annual mean and four season air temperature trend for three periods (1980–2013, 1980–1997, and 1998–2013). For the whole period (1980–2013, Fig. 2a), in terms of annual mean, the Tibetan Plateau and the rest of China have a larger warming amplitude than the global mean (0.16 °C decade^−1^). Meanwhile, similar warming rates are found for four seasons. While the surface air temperature trends of China and the Tibetan Plateau are comparable in spring (March–April–May, MAM) and autumn (September–October–November, SON), and the Tibetan Plateau has a larger warming rate in summer (June–July–August, JJA) and winter (December–January–February, DJF) than other parts of China, making it experiencing a larger warming rate than other parts of China in terms of annual mean temperature. At the first stage (1980–1997, Fig. 2b), other parts of China is characterized by a more rapid warming rate than the Tibetan Plateau, and the most significant warming season is winter for other parts of China. At the second stage (1998–2013, Fig. 2c), during the global climate warming hiatus, other parts of China experience a remarkable cooling trend in winter and spring, leading to annual mean cooling. In contrast, the warming trend remains over the Tibetan Plateau for all seasons, except autumn. Therefore, in contrast to the slowdown of the global warming or the cooling trend in other parts of China, the Tibetan Plateau has experienced a unique accelerated warming trend since 1998.

**Figure 3:**
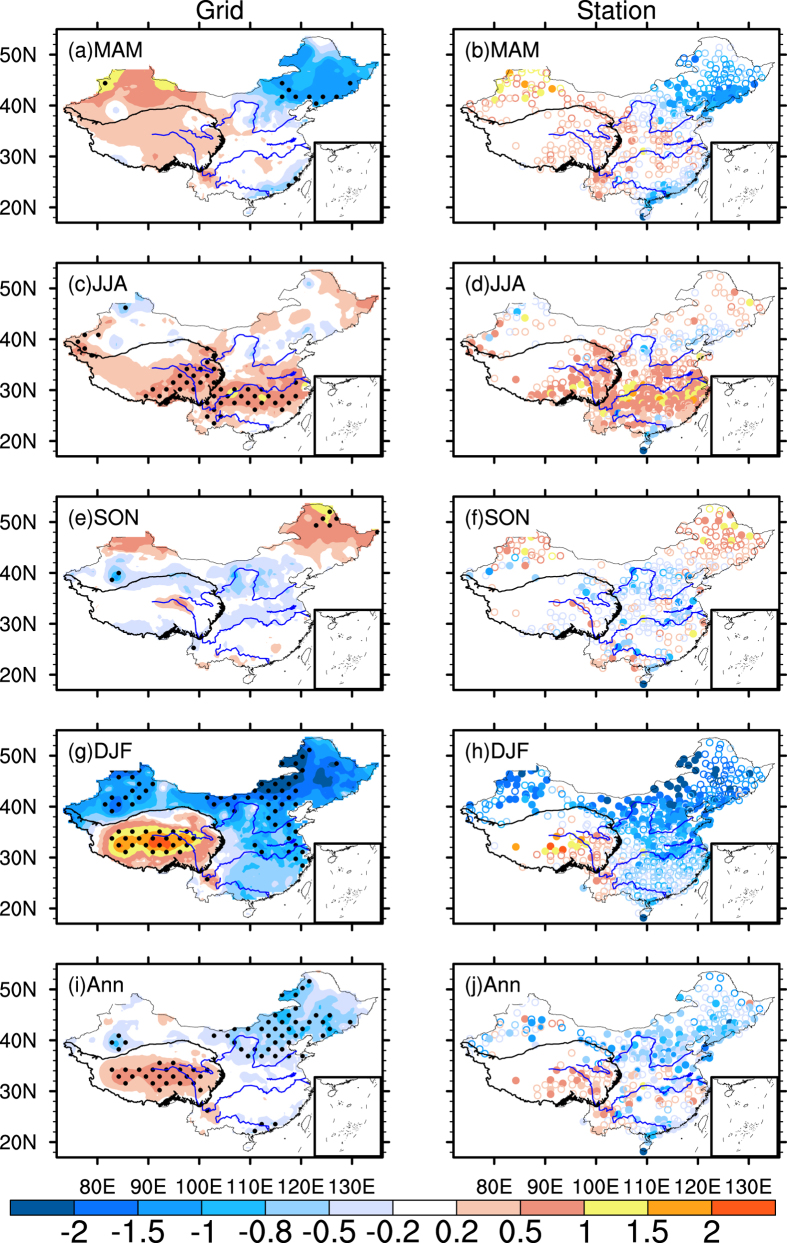
The distributions of the annual and seasonal mean grid temperature (left column) and station temperature (right column) trend during 1998–2013. The dotted areas indicate the trends are significant at 90% confidence level according to a two-tailed Student’s t-test, the same as to the filled circles in the right column. Units: °C decade^−1^. (**a**,**b**) spring (MAM), (**c**,**d**) summer (JJA), (**e**,**f**) autumn (SON), (**g**,**h**) winter (DJF), and (**i**,**j**) annual mean. The figures were produced using NCL.

As suggested by many previous studies^23^,^26^,^27^, the climate warming usually has a non-uniform distribution over China. Figure 3 presents the spatial distribution of the surface air temperature trend within China during 1998–2013, based on both station observations and the gridded data provided by China Meteorological Administration (CMA). It is clear that the Tibetan Plateau is the most warmed area in all seasons except autumn. In spring, the Tibetan Plateau and the regions to its east at the same latitudes share a similar warming trend, while a cooling trend is detected over northeastern and southeastern China (Fig. 3a,b). In summer, most of China, except certain stations over western and northern China, experiences a strong warming trend with a maximum warming rate of 1.97 °C decade^−1^ (Fig. 3c,d). The temperature changes little in autumn and has an opposite trend to spring; i.e., warming over northeastern and southeastern China but cooling over northern and central China (Fig. 3e,f). A sharp temperature trend contrast was found in winter (Fig. 3g,h). The strongest warming centre appears over the Tibetan Plateau, while other parts of China have a dramatic cooling trend. The annual air temperature shows a dramatic warming trend over the Tibetan Plateau and cooling trend in other parts of China (Fig. 3i,j). As shown in Fig. 3, the gridded temperature trend agrees well with the station observed temperature trend.

**Figure 4:**
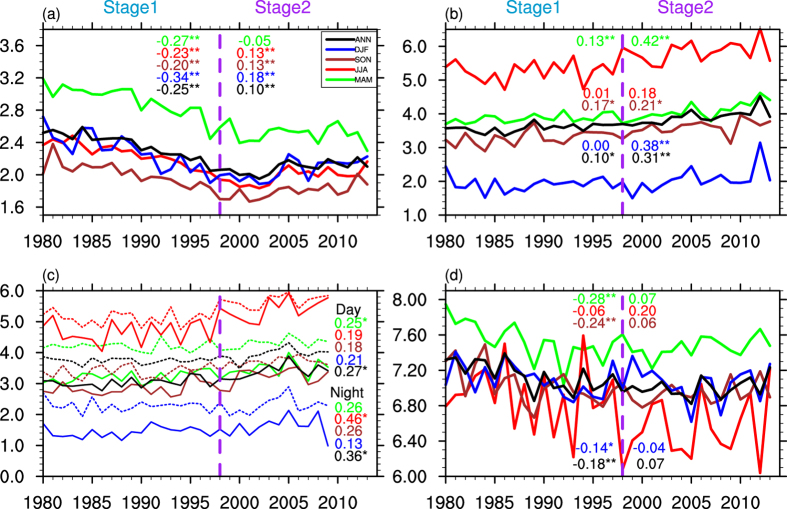
Temporal evolution of the annual and seasonal mean (a) 10 m wind speed (units: m s^−1^), (b) low-level cloud, (c) low-level cloud in daytime (1980–2009, dash lines, 06-18 local time) and nighttime (solid lines, 18–06 local time), only the trends at the second stage are shown here, and (d) sunshine duration (units: h) over the Tibetan Plateau during 1980–2013. The numbers indicate the corresponding trends. Low-level cloud amount varies from 0 to 10 tenths of sky cover. Units for the trend are (**a**) m s^−1^ decade^−1^, (**b**) and (**c**) tenths decade^−1^, and (**d**) h decade^−1^. Label *(**) indicate the trend is significant at 90% (95%) confidence level, according to a two-tailed Student’s *t*-test. The figures were produced using NCL.

Yang *et al*.^28^ demonstrated that the rapid warming over the Tibetan Plateau since the 1980s was a response of regional circulation to global warming. The warming amplitude over high latitudes is larger than that in low latitudes over the Tibetan Plateau, so the weakening of wind speed leads to reduced heat transfer beyond the Tibetan Plateau; thus more energy remains in the Tibetan Plateau, warming local air. Can this mechanism be used to explain the accelerated warming over the Tibetan Plateau during the second stage? In Fig. 4a we plot the temporal variation of the 10 m wind speed over the whole Tibetan Plateau, averaged by stations from 1980–2013, for the four seasons and the annual mean. It is clear that the wind speed features a steady declining trend at the first stage, in accordance with previous findings^28^,^29^. Post-1990s, the *in situ* wind speed recovers gradually, thus the energy concentration cannot explain the accelerated warming trend over the Tibetan Plateau after 1990s. As reported by Duan and Wu^30^, the low-level cloud amount exhibited a significant increasing trend at nighttime during 1961–2003, leading to enhanced nocturnal warming. This factor enhances the warming of the Tibetan Plateau, and the nighttime temperature increase with larger amplitude than daytime temperature, reducing the diurnal temperature range. In Fig. 4b, we see that the low-level cloud amount has increased remarkably since 1998, compared with the first stage. Based on the daytime and nighttime station observed low-level cloud data, we find that the low-level cloud amount increases slightly in the day and at night at the first stage (Fig. 4c). At the second stage, however, the trend is larger during the nighttime than in daytime during 1998–2009, favouring *in situ* atmosphere counterradiation enhanced and rapid warming, especially in summer and annual mean. Actually, the nighttime low-level cloud also shows a larger increase trend than daytime cloud in winter during 1998–2008 (0.51 tenths decade^−1^, significant at 95% confidence level, at nighttime compared with 0.29 tenths decade^−1^ in the daytime) after the abnormal minimal value removed in 2009. Meanwhile, the sunshine duration over the Tibetan Plateau decreases at the first stage, but it increases at the second stage (Fig. 4d), favouring more direct solar radiation absorption during the day.

**Figure 5:**
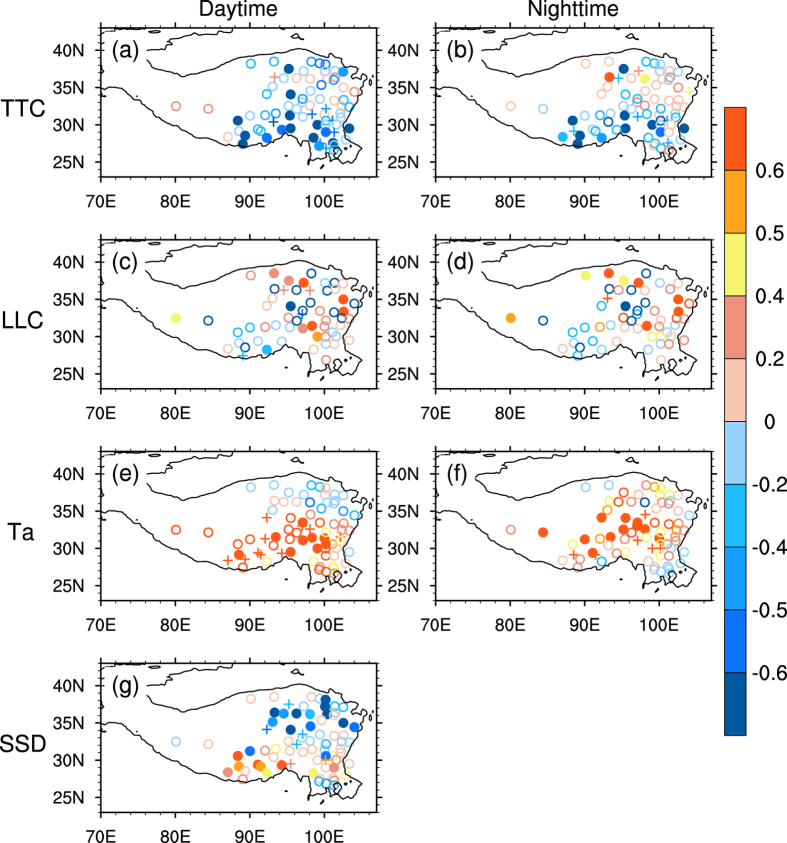
The linear trend of annual mean (a,b) total cloud, (c,d) low-level cloud, (e,f) surface air temperature, and (g) sunshine duration during 1998–2009 in daytime (left column) and nighttime (right column). The crosses (filled circles) indicate the trend is significant at 90% (95%) confidence level, according to a two-tailed Student’s *t*-test. Units for the trends are (**a**–**d**) tenths decade^−1^, (**e**,**f**) °C decade^−1^ and (**g**) h decade^−1^. The figures were produced using NCL.

**Figure 6:**
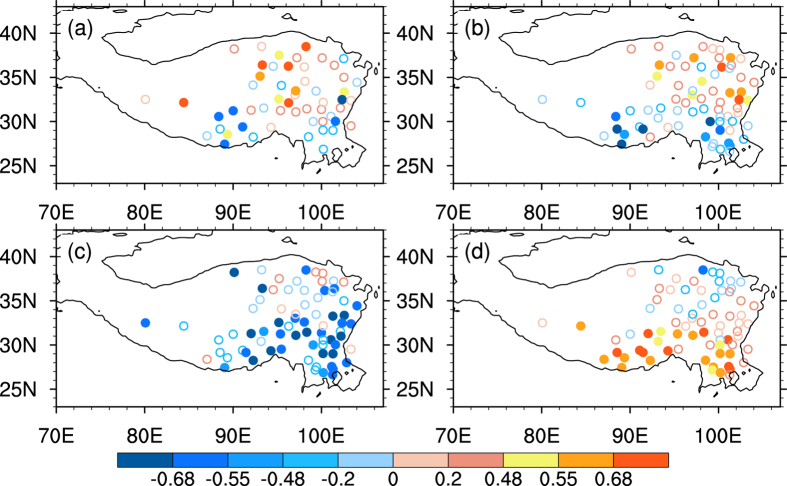
The correlation coefficients between annual mean cloud, surface air temperature and sunshine duration during 1998–2009. (**a**) low-level cloud and surface air temperature at nighttime, (**b**) total cloud and surface air temperature at nighttime, (**c**) total cloud and sunshine duration in daytime, (**d**) sunshine duration and surface air temperature in daytime. 0.48, 0.55 and 0.68 are the thresholds of the significant at 90%, 95% and 99% confidence level according to a two-tailed Student’s *t*-test, shown in filled circles. The figures were produced using NCL.

The climate change shows spatial heterogeneity over the Tibetan Plateau as shown in Fig. 5. Conventionally, a solar radiation trend is explained by aerosol loads and/or cloud changes, while the Tibetan Plateau is one of the regions nearly free from man-made aerosols in the world and it is not expected that a considerable aerosol would affect solar radiation^28^. The total cloud decreases dramatically over the southern Tibetan Plateau (Fig. 5a,b) and a slightly increases over the northern Tibetan Plateau at nighttime (Fig. 5b), resulting the sunshine duration and the daytime surface air temperature increased over the southern Tibetan Plateau, vice versa to the northern Tibetan Plateau (Fig. 5e,g) . Due to both the total cloud and low-level cloud increase at nighttime over the northern Tibetan Plateau (Fig. 5b,d), and the nighttime air temperature shows a weak warming trend there (Fig. 5f) by the cloud heat preservation effect enhanced at nighttime for the cloud cover increases (Fig. 6a,b). The daytime total cloud shows a negative relationship with sunshine duration over the southern Tibetan Plateau (Fig. 6c) and sunshine duration increases, favouring daytime surface air temperature warming (Fig. 5e). Thus the diurnal temperature range decreases over the northern Tibetan Plateau and increases over the southern Tibetan Plateau during the warming hiatus period^31^, due to the non-uniform temperature trend over the Tibetan Plateau.

Therefore, the recent accelerated warming trend over the Tibetan Plateau may be due to the rapid cloud amount increases at nighttime over the northern Tibetan Plateau and the sunshine duration increase in the daytime over the southern Tibetan Plateau.

## Discussion

Based on historical records at 636 meteorological stations, and gridded surface air temperature data across China during 1980–2013, we investigated the recent temperature trend over the Tibetan Plateau. We compared the results with those in other parts of China and the global mean. The results indicate that, in contrast to the cooling trend in other parts of China and the hiatus of climate warming for the global mean since 1998, rapid climate warming still persists over the Tibetan Plateau, which may be related to the increased nighttime cloud amount over the northern Tibetan Plateau and the sunshine duration in the daytime over the southern Tibetan Plateau, whereas the energy concentration due to the decelerated surface wind speed cannot explain the later accelerated warming.

In this paper we proposed a possible mechanism causing the rapidly warming over the Tibetan Plateau. While other factors may play an important role, such as greenhouse gases emissions^32^,^33^, a greater increased in downward longwave radiation at higher elevations in response to the increase of water vapour^34^. The internal climate modes (Atlantic Multidecadal Oscillation and Pacific Decadal Oscillation) may lead to a global warming hiatus^35^, however, the internal variability only explains 18% of the Tibetan Plateau annual mean temperature variance during 1998–2013 based on a multiple regression analysis and the cloud-radiation feedback explains 29%. Furthermore, the cloud-radiation feedback explains 43% of the temperature variance over the southern Tibetan Plateau, southward of 35°N, the most rapidly accelerated warming areas (0.33 °C decade^−1^ compared with 0.25 °C decade^−1^ over the whole Tibetan Plateau during 1998–2013) and the internal variability only explains 17% (see Supplementary Fig. S1 online). The other possible reasons for the accelerated climate warming over the Tibetan Plateau require further assessment in future. As for the recent cooling trend over other parts of China, the question of whether natural variability or the human activity plays the dominant role is still open.

## Methods: Data

We obtained the HadCRUT4 combined land air temperature and SST dataset^36^ with a spatial resolution of 5° × 5° from http://www.cru.uea.ac.uk, including 100 ensemble members from which we sampled the estimated observational uncertainty. The regular daily surface meteorological observations are provided by the China Meteorological Administration (CMA), including air temperature at 2 m above the surface, wind speed at 10 m above the surface, sunshine duration, low-level cloud amount, and 6-hourly total cloud for 636 stations (Fig. 1c), in which 73 stations are in the Tibetan Plateau (Fig. 1c, blue dots). All of the 73 Tibetan Plateau stations have an altitude higher than 2000 m above mean seal level. To obtain more comprehensive information over the Tibetan Plateau, the daily gridded surface air temperature, with a horizontal resolution of 0.5° × 0.5° for the China domain from National Meteorological Information Center^37^ were compared with the station data. All the data used in the study cover the same period of 1980–2013. The seasonal mean surface observed daytime (06–18 local time) and nighttime (18-06 local time) low-level cloud covers are obtained from the Carbon Dioxide Information Analysis Center (CDIAC) Cloud Climatology for Land Station Worldwide (http://cdiac.ornl.gov/) during 1971–2009.

In this paper, a simple linear regression equation is employed here to calculate the trend, i.e. *y*_*i*_=*at*_*i*_+*b*(*i* =1,2,3, …*n*), where y is the climatic variable with a sample size n, parameter t is the corresponding time, a and b are the linear regression coefficient (i.e. Linear Variation Rate) and regression constant. They can be estimated by using the least squares method:


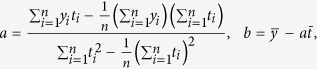


where


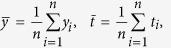


It is worth to note that we obtain similar results from Theil-Sen trend estimation compared with the linear trend used in this paper. And all figures in this paper are created by an open access software NCAR Command Language (NCL, The NCAR Command Language (Version 6.2.1) [Software]. (2014). Boulder, Colorado: UCAR/NCAR/CISL/VETS. http://dx.doi.org/10.5065/D6WD3XH5).

## Additional Information

**How to cite this article**: Duan, A. and Xiao, Z. Does the climate warming hiatus exist over the Tibetan Plateau? *Sci. Rep*. **5**, 13711; doi: 10.1038/srep13711 (2015).

## Supplementary Material

Supplementary Information
