# Supplementary material for: Does the climate warming hiatus exist over the Tibetan Plateau?
Source: Sci Rep. 2015 Sep 2;5:13711. doi: 10.1038/srep13711 (PMC4557067; doi:10.1038/srep13711)
Supplement: Supplementary Information [file srep13711-s1.pdf]

Supplementary Information to

Does the climate warming hiatus exist over the Tibetan Plateau?

Anmin Duan[1, 2*] and Zhixiang Xiao[1, 3]

[1]*State Key Laboratory of Numerical Modelling for Atmospheric Sciences and Geophysical Fluid Dynamics (LASG), Institute of Atmospheric Physics (IAP), Chinese Academy of Sciences (CAS), Beijing 100029, China*

[2]*Collaborative Innovation Center on Forecast and Evaluation of Meteorological Disasters, Nanjing University of Information Science & Technology, Nanjing 210044, China*

[3]*University of Chinese Academy of Sciences, Beijing 100049, China*

Submitted to Scientific Reports

***Corresponding author:**

Dr. Anmin Duan

LASG, Institute of Atmospheric Physics

Chinese Academy of Sciences

P. O. Box 9804

Beijing 100029, China

E-mail: amduan@lasg.iap.ac.cn

Phone: 86-10-8299-5297

Fax: 86-10-8299-5172

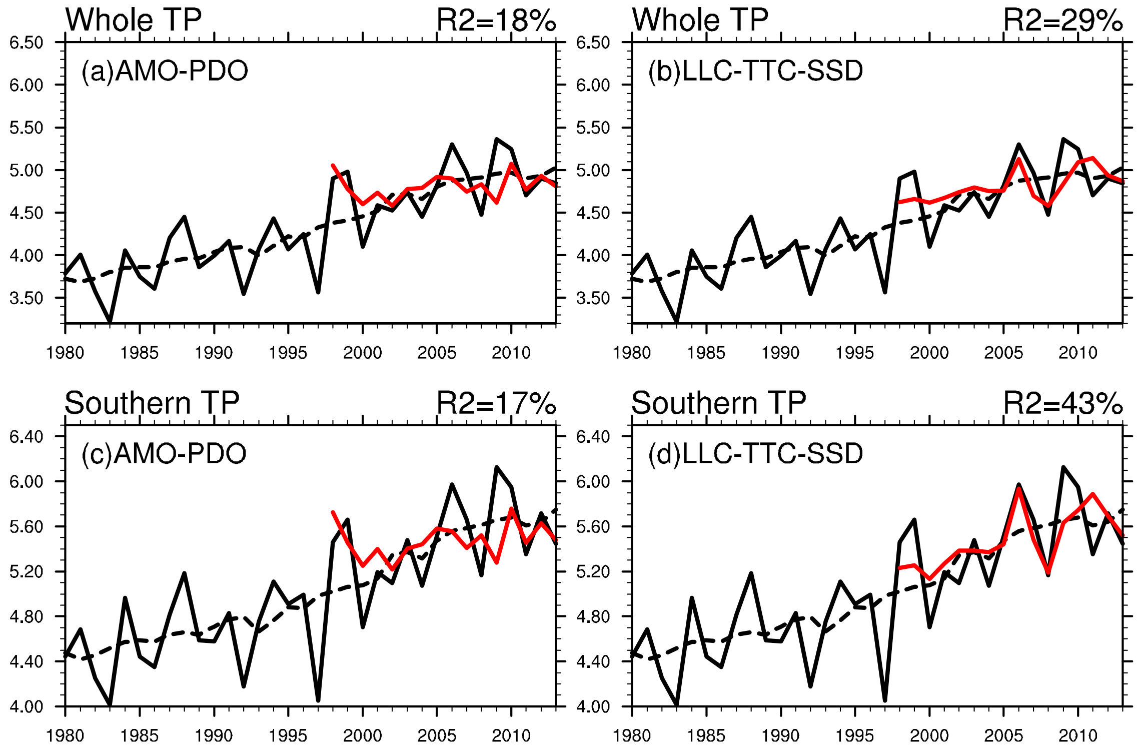

Figure S1. The variation of annual mean surface air temperature (Units: ℃) over the Tibetan Plateau (a-b, black lines, dash lines indicate the 9 year running mean) and southern Tibetan Plateau (c-d, southward from 35°N). The red lines indicate the temperature regresses by Atlantic Multidecadal Oscillation and Pacific Decadal Oscillation (a and c) during 1998-2013, and low-level cloud, total cloud and sunshine duration (b and d). The explained variance is shown at the upper right corner of each panel.
